# Overexpression of *OsSWEET5* in Rice Causes Growth Retardation and Precocious Senescence

**DOI:** 10.1371/journal.pone.0094210

**Published:** 2014-04-07

**Authors:** Yong Zhou, Li Liu, Weifeng Huang, Meng Yuan, Fei Zhou, Xianghua Li, Yongjun Lin

**Affiliations:** 1 National Key Laboratory of Crop Genetic Improvement and National Centre of Plant Gene Research, Huazhong Agricultural University, Wuhan, China; 2 Plant Reproductive Biology, University of California Davis, Davis, California, United States of America; New Mexico State University, United States of America

## Abstract

As a novel sugar transporter family, SWEETs play important roles in plant growth and development. Here, we characterized a SWEET gene named *OsSWEET5* through its overexpression in rice. Heterologous expression assay indicated that *OsSWEET5* encoded a galactose transporter in yeast. *OsSWEET5*-overexpressing plants displayed the phenotypes of growth retardation and precocious senescence at seedling stage. GC-MS analysis showed that the sugar levels were largely altered in the leaves of the *OsSWEET5*-overexpressing plants. Molecular analysis revealed that these phenotypes might be due to the transcriptional changes of the genes involved in sugar metabolism and transport. In addition, the transgenic plants showed a lower level of auxin with altered transcription of genes involved in auxin signaling and translocation pathways. However, no obvious phenotype was observed between the *amiRNA*-*OsSWEET5* transgenic lines and WT plants, which could be a result of the functional redundancy of the galactose transporters in rice. Taken together, our findings suggest that OsSWEET5 plays a crucial role in regulating the crosstalk between sugar and auxin in rice.

## Introduction

Plants absorb solar energy during photosynthesis for fixation of carbon in leaves and produce photoassimilates, which are transported into storage pools such as plastidic starch or vacuolar sugars. The transportation and distribution of photoassimilates in higher plants from phototrophic to heterotrophic cells and tissues depend on the activities of numerous transporters [1]. Carbohydrates are transported from the source to photosynthetically inactive sink tissues mainly in the form of sugar, especially sucrose. Cell-to-cell transport of sucrose depends on the activities of plasma-membrane-located sucrose transporters (SUTs) [2]. And sucrose can be hydrolysed by cell-wall-bound invertases to glucose and fructose, which can be transported into cells via monosaccharide transporters (MSTs) [3]. Normal transportation of sugars from source leaves to sink tissues or organs is very important for plant growth and development [4]. Imbalanced carbohydrate distribution between source and sink at the whole plant level can cause decreased expression of photosynthetic genes, and reduce the growth rate of the plant [5]. For example, ZmSUT1 functions in phloem load sucrose in maize leaves, and the mutants of *ZmSUT1* hyperaccumulated soluble sugars in leaves, displaying the phenotypes of leaf chlorosis and reduced growth [6], [7]. OsSUT2 is involved in sucrose transport across the tonoplast from the vacuole lumen to the cytosol in rice, and *ossut2* showed obviously increased levels of sucrose, glucose and fructose compared with the controls, leading to a phenotype of growth retardation [8]. AtSWEET17 is the first vacuolar fructose transporter, which can export fructose out of the vacuole; and *AtSWEET17* mutations caused stunted growth and affected seed yield, suggesting that AtSWEET17 can control the fructose level of leaf in Arabidopsis [9]. However, the mechanism of the source-sink interaction for sugar transport remains elusive.

Many studies have demonstrated a potential link between sugar and auxin signaling pathways [10], [11], [12], [13]. On one hand, auxin can regulate sugar synthesis and transport in plants. For example, OsSAUR39 acts as a negative regulator of auxin synthesis and transport in rice, and overexpression of *OsSAUR39* in rice caused sugar accumulation and transcriptional changes of the genes involved in sugar synthesis and transport [14], [15]. In tomato, a member of auxin response factor (*ARF*) gene family named *SlARF4* plays a major role in mediating the auxin control of sugar metabolism during fruit development [16]. On the other hand, as signaling molecules, sugars play central roles in regulating the expression of auxin-responsive genes to modulate auxin biosynthesis and signaling. For example, sugar levels can regulate the transcript of *ZmYUC* to modulate the tryptophan-dependent auxin biosynthesis in developing maize kernels [12]. A previous study also has showed that IAA biosynthesis is regulated by endogenous sugar levels [17]. And it has been reported that the control of glucose to root growth and development in Arabidopsis is probably through auxin signaling [13]. In addition, some studies have suggested that auxin-induced growth can be inhibited by galactose [18], [19], [20], and this inhibition may be due to the inhibition of auxin-induced H^+^-excretion needed for the initiation of rapid elongation or the inhibition of cell wall synthesis [21].

There are a number of sugar transporters which are involved in galactose transport and play important roles in many physiological pathways in plants. CkHUP2 (*Chlorella kessler* hexose uptake 2) is a high-affinity transporter for galactose in *Chlorella kessleri*, which may function in the import of organic compounds during the heterotrophic growth of plant cells [22]. OsMST4, a functional monosaccharide transporter capable of transporting galactose, is expressed in both the source and sink tissues, and is suggested to participate in many developmental stages in rice [23]. In Arabidopsis, the sugar transport protein AtSTP1, a high-affinity H^+^-monosaccharide symporter which can transport galactose, plays important roles in the uptake of extracellular sugars by the embryo and in seedlings [24], [25]. A subsequent research found that *AtSTP1* is expressed in guard cells and has a role in the import of monosaccharide into guard cells during night [26]. AtSTP11, which is exclusively expressed in pollen tubes, is another high affinity hexose-specific H^+^-symporter involved in galactose transport, and plays a role in the supply of monosaccharides to growing pollen tubes [27]. AtSTP14 is the first plant transporter specific for galactose and is suggested to be involved in the retrieval of cell wall-derived galactose; meanwhile its expression is regulated by darkness, sugar starvation, senescence and drought stress, which eventually induce cell wall degradation [28]. AtSTP2 also could serve in the uptake of galactose into the developing male gametophyte, and galactose is proposed to be a degradation product from cell-wall components [29]. Therefore, it can be speculated that galactose transporters probably participate in cell wall galactose recycling or/and galactose supplying to tissues or organs during different developmental stages in plants.

The MtN3/saliva proteins were first described in *Medicago truncatula* [30]. Recent studies have shown that many members of MtN3/saliva family belong to the SWEET subfamily and can mediate sugar transport, and the sugar transporters probably supply carbohydrates to various tissues in both monocots and dicots [31], [32]. For example, OsSWEET11 and OsSWEET14 not only mediate the glucose import and efflux in human embryonic kidney (HEK) 293T cells and *Xenopus* oocytes, but also serve as low-affinity sucrose transporters [31], [33]. *OsSWEET11* showed high expression levels in panicles and anthers, and *OsSWEET11*-RNAi transgenic plants showed a severely reduced fertility and even complete sterility, suggesting that OsSWEET11 plays an important role in regulating the reproductive development of rice [32], [34]. *OsSWEET14* homozygous T-DNA insertion mutant plants showed remarkable delayed growth compared with the heterozygous mutant plants [35]. In Arabidopsis, *AtSWEET11* and *AtSWEET12* function as low-affinity transporters for the efflux of sucrose from phloem parenchyma cells into the apoplast, and single *AtSWEET11* or *AtSWEET12* mutants showed no visible phenotype, but the *atsweet11atsweet12* double mutant plants displayed the phenotype of reduced growth [33]. AtSWEET17 acts to export fructose out of the vacuole and has a role in carbohydrate partitioning in plants, which can regulate the developmental processes of plants [9]. AtSWEET16 can catalyze the transport of glucose, fructose, and sucrose, and *AtSWEET16*-overexpressing plants displayed significant alterations in sugar levels as well as in different development processes like germination, growth, and stress tolerance [36]. Therefore, SWEETs may be key regulators in plant growth and development.

In this paper, we characterized a member of SWEETs named *OsSWEET5* in rice, which encoded a sugar transporter protein involved in galactose transport. The *OsSWEET5*-overexpressing plants showed retarded growth in the early seedling stage with altered sugar metabolism and transport as well as inhibited auxin signaling and translocation. The study was aimed to achieve a better understanding on the possible physiological functions of OsSWEET5 and thus to optimize the transport and reserve of carbohydrates to raise the yield of rice.

## Materials and Methods

### Plant materials and growth conditions

Zhonghua 11 (*Oryza Sativa* L. ssp. *Japonica* cv. Zhonghua 11) was used in this study. *OsSWEET5* transgenic plants and Zhonghua 11 (as the wild type) were planted in the field of Huazhong Agricultural University (Wuhan, China).

### Plasmid construction and rice transformation

For *P_OsSWEET5_*::GUS vector construction, *OsSWEET5* promoter (about 2.3 kb 5′-upstream fragment of *OsSWEET5*) was amplified using rice genomic DNA as a template. The DNA fragment was inserted into the pDX2181 vector [37]. For overexpression vector construction, the DNA fragment was amplified, digested with *Kpn* I and *Xba* I and ligated to the pCAMBIA1300 under the control of the 35S promoter. Constructs were transformed into Zhonghua 11 as previously described [38]. The independent *OsSWEET5*-overexpressing transgenic plants were further confirmed by PCR assay and Southern blot [38]. The method of Southern blot was also described in Methods S1. The PCR program was as follows: 94°C for 5 min, followed by 30 cycles of 94°C for 1 min, 58°C for 1 min, and 72°C for 1 min, 72°C for 7 min. The sequences of primers are listed in Table S1.

### Histochemical assay

The histochemical GUS assay was performed as previously described [37]. Samples from independent *P_OsSWEET5_*::GUS transgenic plants were incubated at 37°C in GUS reagent for about 10 h after 15 min vacuum filtration. All the samples were destained by 75% (v/v) alcohol and observed subsequently using a dissecting microscope (Leica, Germany).

### Subcellular localization

The ORF of *OsSWEET5* with the exception of stop codon was amplified using the full-length cDNA clone J023023E05 (http://cdna01.dna.affrc.go.jp/cDNA) as a template and cloned into the pM999-35S-EGFP vector. Rice protoplasts transformation was performed as described earlier [39]. GFP fluorescent signals were observed and photographed using CLSM (Leica, Germany) after 20 h of dark culture at 28°C.

### Functional characterization of OsSWEET5 in yeast

Yeast plasmids were kindly provided by Dr. Eckhard Boles, Johann Wolfgang Goethe-Universität Frankfurt, Germany and the vector backbones p426 and p413 were constructed as previously described [40]. The *HXT7* (*hexose transporter 7*) promoter was amplified by using vector p426-pHXT7-HXT7 as template and cloned into vector p413GPD to form p413-pHXT7 as negative control. The *HXT7* gene was amplified by using vector p426-pHXT7-HXT7 as template and inserted into p413-pHXT7 to form p413-pHXT7-HXT7 as positive control. The ORF of *OsSWEET5* was amplified and cloned into p413-pHXT7, yielding construct p413-pHXT7-OsSWEET5. These constructs were transformed into a hexose transport-deficient yeast strain EBY.VW4000 (*MATα Δhxt1-17 Δgal2 Δstl1 Δagt1 Δmph2 Δmph3 leu2-3,112 ura3-52 trp1-289 his3-Δ1 MAL2-8^c^ SUC2*) [41] and grown on synthetic deficient medium containing either 2% maltose (as control) or 2% glucose, 2% fructose, 2% mannose, 2% galactose, 2% sucrose, respectively. Due to the lack of all the *HXT* genes, EBY.VW4000 no longer grows on monosaccharides but can grow on maltose. Synthetic medium consisted of 6.7 g/l Difco yeast nitrogen base (YNB) supplemented with essential amino acids. The transformants were then grown overnight in liquid minimum medium to OD_600_ = 1.0. A 4 μl aliquot of the transformants and each of the three consecutive 1/10 dilutions (0.1, 0.01, and 0.001) were spotted and incubated for 3–5 days at 30°C.

### RNA extraction, RT-PCR, qRT-PCR and Northern blot analysis

Total RNA was isolated using TRIzol reagent (Invitrogen, Carisbad, CA, USA) according to the manufacturer's instruction. First-strand cDNAs were synthesized from RNase-free DNase I-treated (Invitrogen, USA) total RNA to eliminate genomic DNA contamination according to the manufacturer's instruction. The RT-PCR program was as follows: 94°C 3 min, followed by 28–30 cycles of 94°C for 1 min, 58°C for 1 min, and 72°C for 30 s, and then a final extension at 72°C for 7 min. Rice *Actin1* was used as an internal control. qRT-PCR was performed with Applied Biosystems 7500 Real-Time PCR System (Applied Biosystems, Carlsbad, CA, USA) using SYBR Green I (TaKaRa, Japan) as previously described [42]. Three replicates were performed for the analysis of each gene. Rice *Actin1* as an internal control and the relative expression levels of target genes were determined as described previously [43]. The sequences of the primers are listed in Table S1. For Northern blot analysis, 15 μg of total RNA was separated in 1.2% (w/v) agarose gel, transferred to Hybond-N nylon membrane (Amersham, USA), and hybridized with a PCR fragment labeled with [α-^32^P] dCTP using a Random Primer DNA Labeling Kit (TaKaRa, Japan). The hybridization signals were obtained by autoradiography in Fujifilm FLA-5100 (Fujifilm, Japan) according to the protocol described previously [44]. The probe for Northern blot was amplified using PCR with primers listed in Table S1.

### Measurement of chlorophyll, auxin and sugar content

Chlorophyll content measurement was carried out according to a previous study [45]. Auxin was extracted and quantificated as described previously [46]. For measurement of sugars, leaves from OX2 and WT were first ground using PBS buffer (pH 7.0) after being weighed. After centrifugation and resuspension in PBS buffer (pH 7.0), samples mixed with internal standard (inositol, Sigma, USA) were dried with dry N_2_ gas and derivatized using N,O-bis[trimethylsilyl]-trifluoroacetamide (Alpha, USA): dimethylformamide (Sigma, USA) (BSTFA: DMF, 1∶1, v/v). Afterwards, the derivatized sample extracts were diluted with acetone and analyzed by GC-MS (SHIMADZU GCMS-QP2010 Plus) with HP-5 MS column (60 m×0.32 mm×0.25 μm) using the methods described previously [47], [48] with modifications. The quantification of sugars was performed with internal standard method, and the retention times were as follows: galactose, 13.7 min and 14.75 min; sucrose, 27.466 min; glucose, 14.43 min and 16.684 min; fructose, 12.355 min, 12.545 min and 12.629 min; and inositol, 19 min.

## Results

### Sequence analysis of OsSWEET5 in rice

*OsSWEET5* (TIGR ID: LOC_Os05g51090) consisting of four exons and three introns and encoding a protein with 237 amino acids was cloned from rice (cv Zhonghua 11) with an ORF of 714 bp. Phylogenetic analysis showed that *OsSWEET5* was a member of SWEETs Clade II subfamily [31]. The predicted OsSWEET5 protein contained two MtN3/saliva domains in the N- and C-terminal regions, which was in accordance with the characteristics of the MtN3/saliva family (Figure S1).

### Expression pattern of OsSWEET5

**Figure 1:**
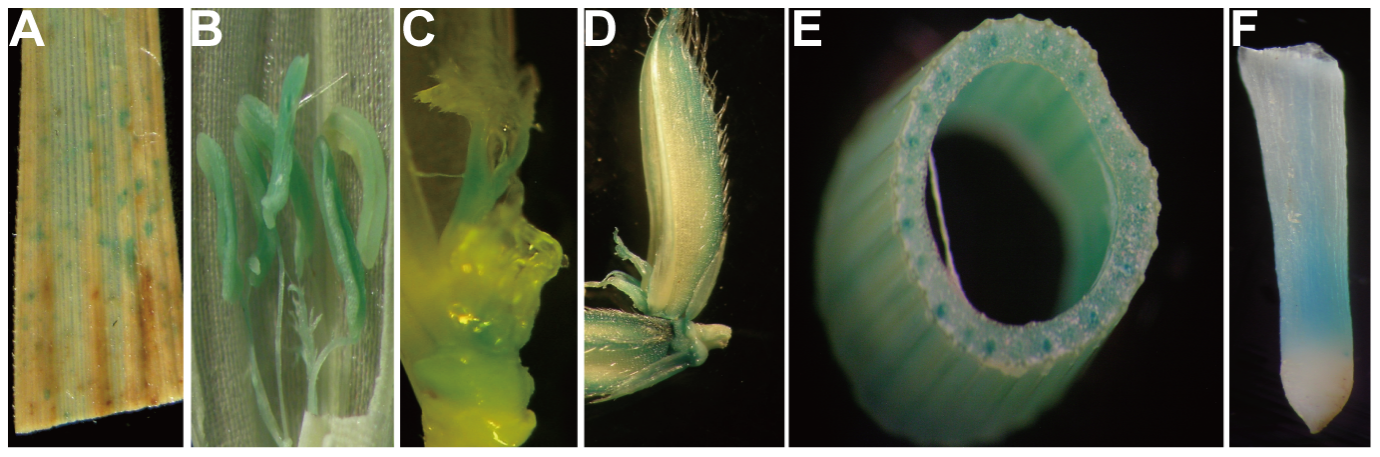
Histochemical localization of *GUS* expression in *P_OsSWEET5_*::GUS transgenic rice. (A) flag leaf at 40 days after heading; (B) stamen; (C) pistil; (D) hull; (E) stem; (F) root at flowering stage.

qRT-PCR analysis revealed that *OsSWEET5* was mainly expressed in the floral organs at the heading stage, and was also detectable in stem, root and senescing leaves (Figure S2). To further examine the spatiotemporal expression pattern of *OsSWEET5*, we generated transgenic rice plants with the *P_OsSWEET5_*::GUS construct and checked the *GUS* activity in five independent transgenic plants. The results showed that *GUS* expression was detected in the senescing leaves, stamen, pistil, hull, stem and root (Figure 1).

### Subcellular localization of OsSWEET5

**Figure 2:**
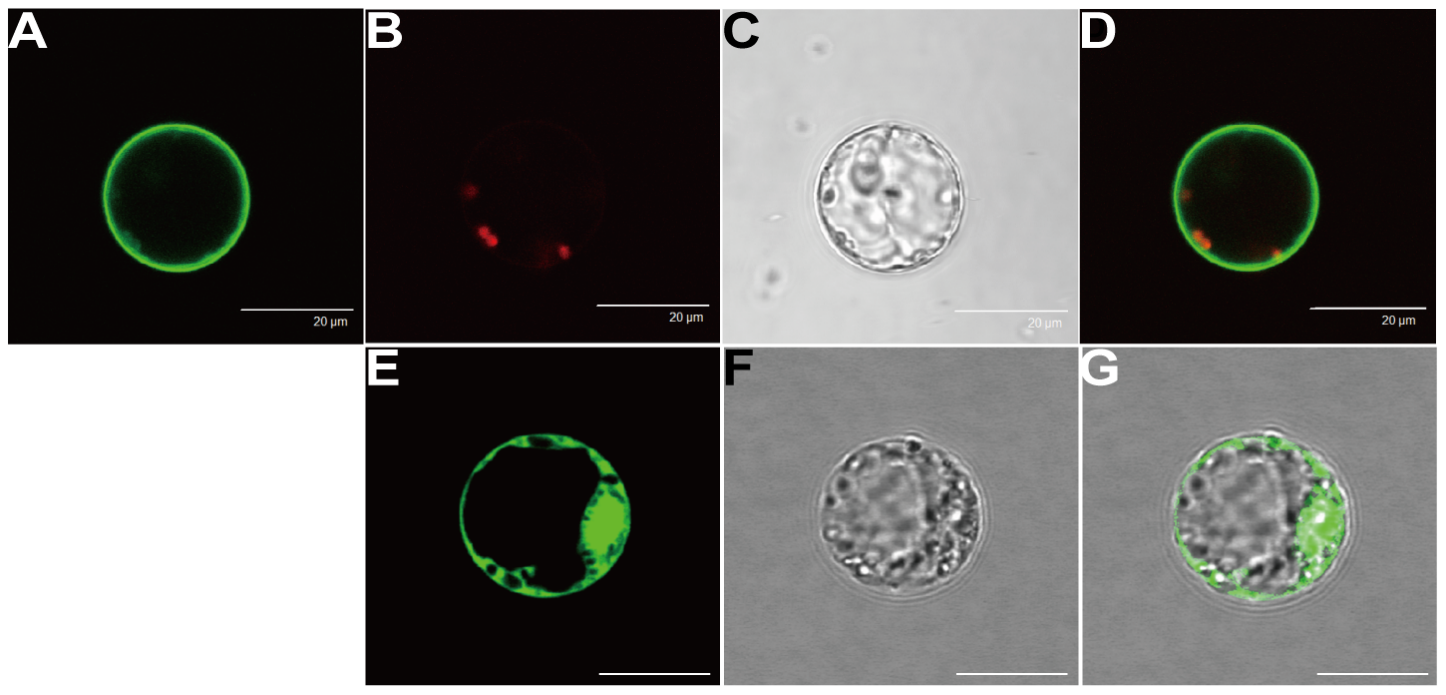
Subcellular localization of OsSWEET5 in rice cell protoplasts. Rice cell protoplasts were transformed using 35S::OsSWEET5-GFP (A–D) and 35S::GFP (E–G). (B) red autofluorescence signals. (C) and (F) bright field. (D) and (G) merged image. 35S::GFP was transformed as a control. The bar indicates 20 μm.

Most of SWEETs are small proteins which are predicted to have seven transmembrane helices, with the first and last 3-transmembrane-helix-domain polypeptide fused via the middle transmembrane helix to form a 3+1+3 configuration structure [31], [32]. In the present study, bioinformatic analyses using TMHMM (http://www.cbs.dtu.dk/services/TMHMM/) and SOSUI (http://bp.nuap.nagoya-u.ac.jp/sosui/) predicted that OsSWEET5 contained seven transmembrane helices (Table S2, Figure S3) [49], [50]. The subcellular localization of OsSWEET5 was then analyzed by transient expression of an OsSWEET5-GFP fusion protein in rice protoplast. Confocal scanning laser microscopy showed that GFP signals were observable at the plasma membrane in OsSWEET5-GFP fusion vector (Figure 2A–D); whereas the GFP control vector displayed fluorescence in the cytosol and nuclei in the cells (Figure 2E–G), which suggested that *OsSWEET5* encoded a plasma membrane protein.

### Sugar transport ability of OsSWEET5 in yeast

**Figure 3:**
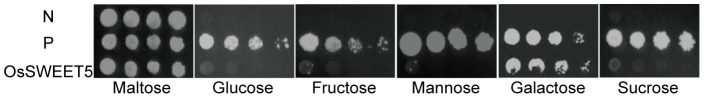
OsSWEET5 had sugar transporter activity involved in galactose. Growth complementation of the yeast mutant strain EBY.VW4000 was restored by OsSWEET5 on the culture medium containing galactose. N, negative control; P, positive control; OsSWEET5, p413-pHXT7-OsSWEET5.

SWEETs have been shown to mediate the sugar transport in *Arabidopsis thaliana* and *Oryza sativa* as uniporters which do not require a proton gradient [31], [33], [51]. To check whether it was involved in sugar transport, *OsSWEET5* was expressed in yeast. The growth of the mutant strain was restored only on the culture medium containing galactose but not glucose, fructose, mannose, or sucrose (Figure 3), suggesting that OsSWEET5 was involved in galactose transport.

### Phenotypes resulting from overexpression of OsSWEET5 at seedling stage

**Figure 4:**
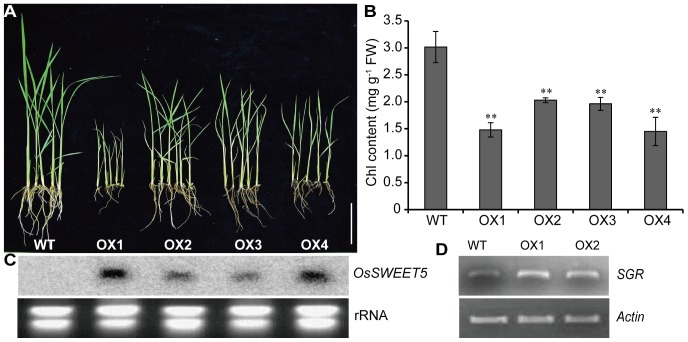
Phenotype and physiological characterization of *OsSWEET5*-overexpressing transgenic plants. (A) Photographs of WT and *OsSWEET5*-overexpressing lines (T_2_) at 15 days after germination. The bar indicates 10 cm. (B) Measurement of chlorophyll content in *OsSWEET5*-overexpressing lines and WT plants. The samples were from the second leaves in (A). The results shown are the means of three independent measurements. Significant differences are calculated by *t*-test and shown by asterisks. *, *P*<0.05 or **, *P*<0.01. FW, Fresh weight. (C) Northern blot analysis of *OsSWEET5*-overexpressing lines. RNA was extracted from the second leaves in (A). (D) RT-PCR analysis of *SGR* in *OsSWEET5*-overexpressing lines (OX1 and OX2) and WT plants. The first-strand cDNAs were prepared using RNAs extracted from the second leaves of OX1, OX2 and WT plants at three-leaf stage.

To further explore the function of *OsSWEET5* in rice, *OsSWEET5*-overexpressing plants were generated and single-copy transgenic plants were confirmed by Southern blot (Figure S4). Most of the transgenic plants showed a phenotype of growth retardation. Four homozygous transgenic lines in T_2_ generation were further analyzed. The results showed that the plant height and root length of the overexpression lines were markedly lower than that of WT plants at seedling stage, indicating a phenotype of growth retardation (Figure 4A). In addition, the chlorophyll levels in transgenic lines were significantly lower than those in WT plants (Figure 4B). Further, Northern blot analysis showed varying degrees of increased transcript abundance of *OsSWEET5* in the leaves of the transgenic plants (Figure 4C). Since *SGR* is a senescence-specific gene in rice and can be used as a molecular marker for leaf senescence [52], [53], the expression of *SGR* was examined. As shown in Figure 4D, the expression levels of *SGR* were higher in the leaves of OX1 and OX2 than in that of WT plants. These findings revealed that the overexpression of *OsSWEET5* caused growth retardation and precocious senescence in rice, and the phenotypes were positively correlated with the expression levels of *OsSWEET5*. A homozygous positive line (OX2) with less growth retardation was selected to further explore the reasons for the abnormal phenotypes.

### Abnormal sugar metabolism and transport in OsSWEET5-overexpressing plants

**Figure 5:**
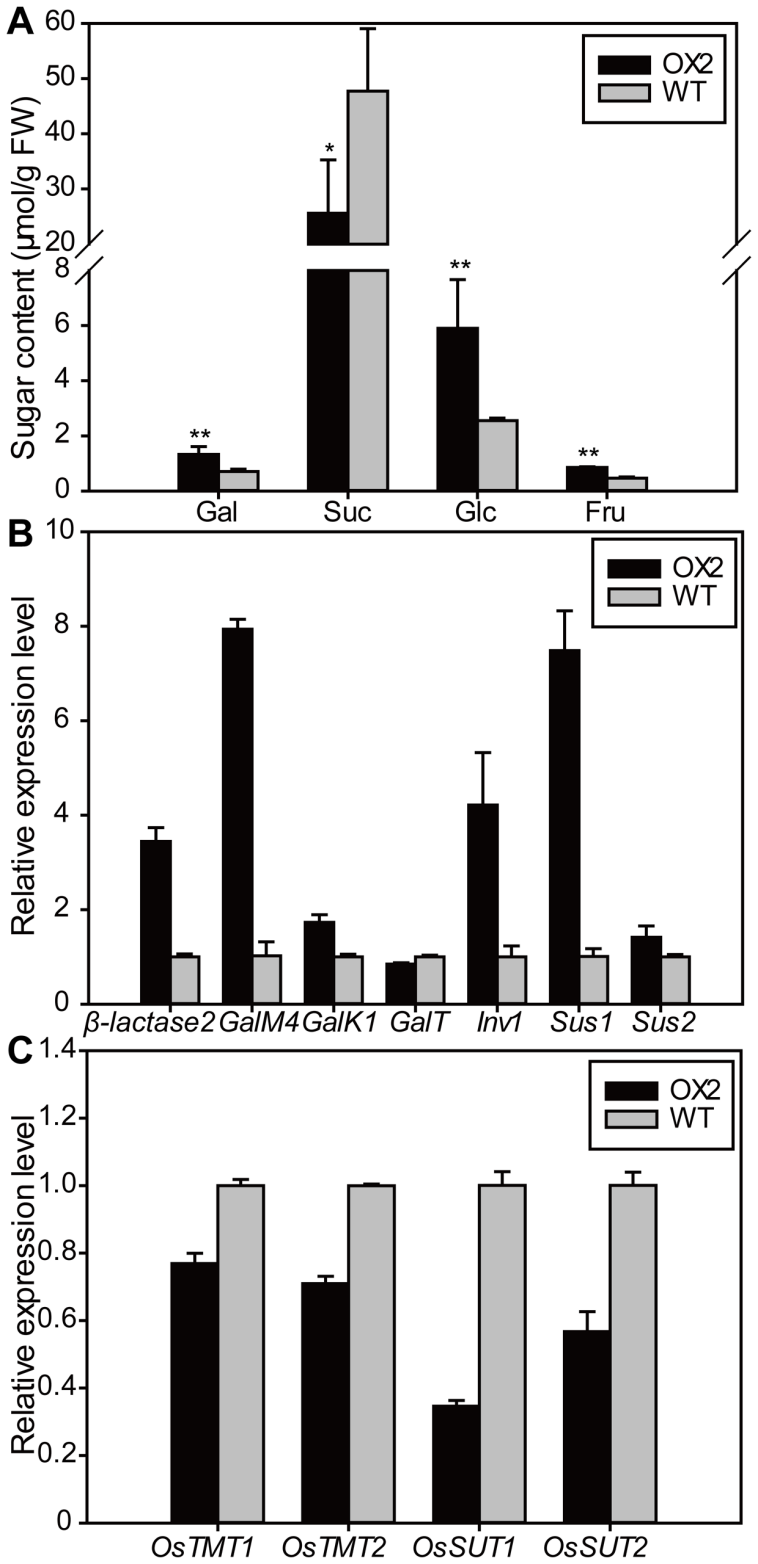
Sugar metabolism and transport were disordered in *OsSWEET5*-overexpressing plants. (A) Sugar levels in leaves of OX2 and WT at three-leaf stage at the end of the light periods. Gal, galactose; Suc, sucrose; Glc, glucose; Fru, fructose. Statistical significance is indicated by * (*P*<0.05) and ** (*P*<0.01) (*t*-test, *n* = 3). FW, Fresh weight. (B) Expression analysis of key genes involved in sugar metabolism in OX2 and WT plants. The first-strand cDNAs were prepared using RNAs extracted from the second leaves of OX2 and WT plants at three-leaf stage. c qRT-PCR analysis of genes involved in sugar transport in OX2 and WT plants. The first-strand cDNAs were prepared using RNAs extracted from the second leaves of OX2 and WT plants at three-leaf stage.

It was shown that OsSWEET5 was involved in galactose transport in yeast (Figure 3). Therefore, the phenotypes of *OsSWEET5*-overexpressing plants may be due to the imbalance of galactose distribution. To verify this, galactose levels in the leaves of OX2 and WT at three-leaf stage were analyzed. The result showed that the galactose level was significantly higher in OX2 leaves than in WT leaves (Figure 5A). To understand the cause of the galactose accumulation, the expression levels of the genes involved in galactose metabolism were examined using qRT-PCR. As shown in Figure 5B, in the OX2 plants, the expression levels of *β-lactase2*, *GalM4* and *GalK1* were much higher than those in the WT plants, whereas the expression of *GalT* was slightly lower. β-Lactase (encoded by *β-lactase2*) is involved in the breakdown of polysaccharide to generate free β-D-Gal, which is converted to α-D-Gal by galactose mutarotase (encoded by *GalM*) [54], [55]. And then α-D-Gal is phosphorylated by galactokinase (encoded by *GalK*) and converted to UDP-Gal by α-D-galactose-1-phosphate uridylyltransferase (encoded by *GalT*) for further metabolism [55], [56]. The qRT-PCR result indicated that the galactose metabolism was changed, which could have impaired the galactose distribution.

To investigate whether the levels of other sugars were altered, sucrose, glucose and fructose levels were also evaluated. Similar to the level of galactose, the levels of glucose and fructose in the leaves of OX2 were enhanced to about 2-fold higher than in those of WT, whereas the level of sucrose was significantly decreased (Figure 5A). To reveal the reasons for the changes, we further analyzed the expression of sucrose cleavage genes using qRT-PCR. As shown in Figure 5B, the transcripts of *Inv1*, *Sus1* and *Sus2* were more obviously increased in OX2 than in WT, indicating that the degradation of sucrose was accelerated in OX2. To check whether the sugar transport was changed or not, we investigated the expression levels of the genes involved in sugar transport by qRT-PCR. As shown in Figure 5C, compared with WT plants, OX2 displayed dramatically reduced transcripts of *OsTMT1*, *OsTMT2*, *OsSUT1* and *OsSUT2* (Figure 5C), suggesting that sugar transport was altered in OX2 compared with in WT plants.

### Inhibition of auxin signaling and translocation in OsSWEET5-overexpressing plants

**Figure 6:**
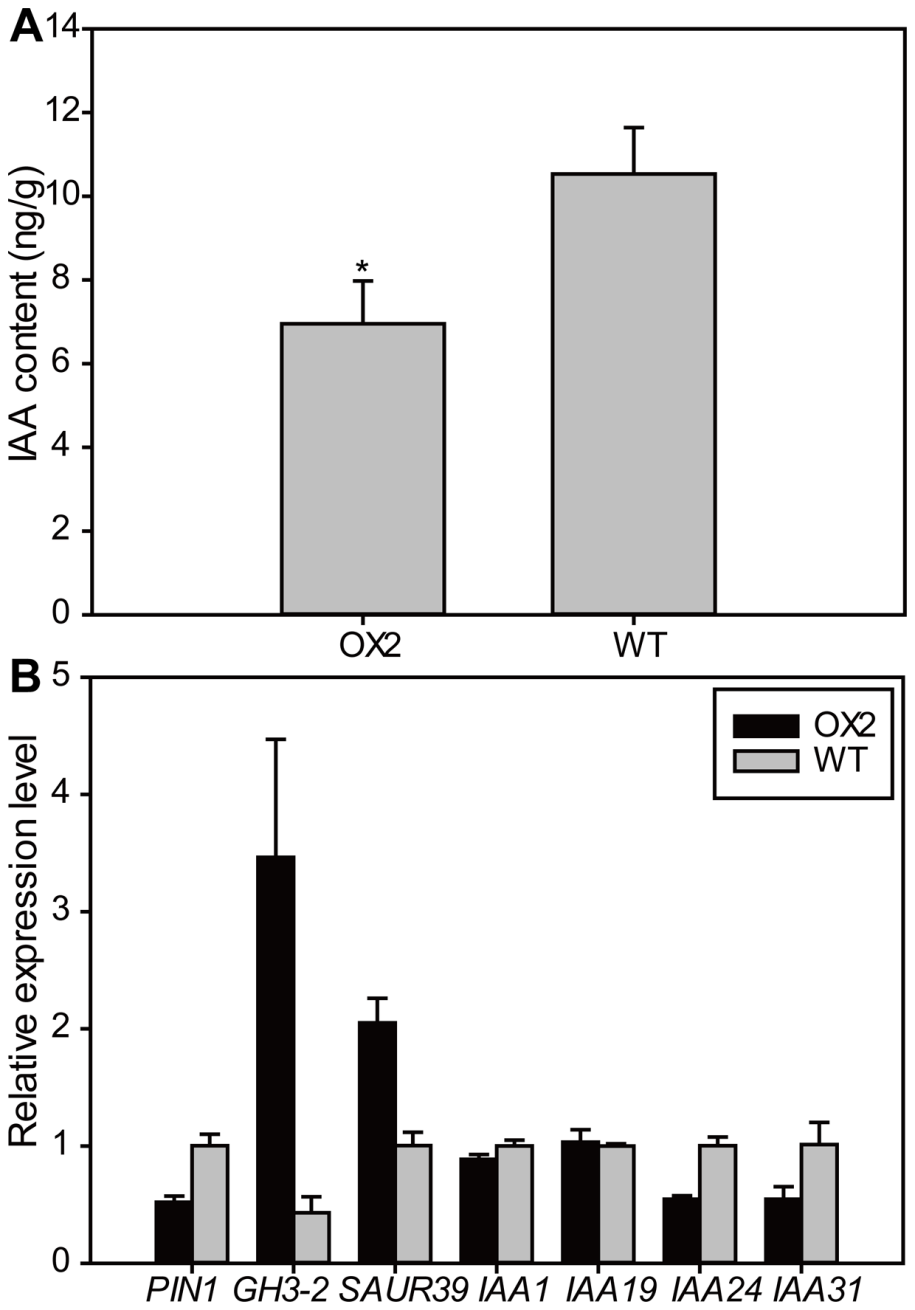
Auxin signaling and translocation were inhibited in *OsSWEET5*-overexpressing line (OX2) compared to WT plants. (A) Quantification of free IAA contents in the second leaves of OX2 and WT plants at three-leaf stage. Values are means ±SD (*n* = 3). Statistical significance is indicated by * (*P*<0.05, *t*-test). (B) Expression levels of auxin-regulated genes in the leaves of OX2 and WT plants using qRT-PCR. The first-strand cDNAs were prepared using RNAs extracted from the second leaves of OX2 and WT plants at three-leaf stage.

Galactose has long been known to be toxic to plant cell and lead to growth retardation through the inhibition of auxin signaling and translocation [18], [21], [57]. To understand the causes of the phenotypes in *OsSWEET5*-overexpressing plants, we evaluated the IAA levels in the transgenic line (OX2) and WT plants at seedling stage. As shown in Figure 6A, the free IAA level was significantly reduced in the leaves of OX2 compared with in those of WT. The lower free IAA level may explain why the transgenic plants exhibited low-auxin phenotypes including dwarfing and the reduction in shoot and root length (Figure 4A).

To investigate the reasons for the decreased IAA level in transgenic plants, we further examined the expression levels of auxin-regulated genes in the leaves of OX2 and WT plants using qRT-PCR. As shown in Figure 6B, the expression levels of *OsGH3-2* and *SAUR39* were markedly increased in OX2 compared with in WT. In addition, the transcripts of *OsPIN1*, *IAA24* and *IAA31* were sharply reduced in *OsSWEET5*-overexpressing plants (Figure 6B). Taken together, these results suggested that auxin signaling and translocation were inhibited in OX2 plants.

## Discussion

### OsSWEET5 is involved in galactose transport in rice

Many SWEETs have been proven to have sugar transport ability and function as facilitators that support both the import and the efflux of sugar into and out of cells [31], [33], [58]. However, OsSWEET5 could catalyze the transport of galactose but not that of glucose, fructose, mannose and sucrose when expressed in yeast (Figure 3), which is different from other published SWEETs [9], [31], [33], [36]. This result suggests that OsSWEET5 plays different roles in rice.

Analysis of the *GUS* expression patterns in *P_OsSWEET5_*::GUS transgenic plants revealed that *OsSWEET5* was expressed in senescing leaves (Figure 1), which suggests the possibility that OsSWEET5 participates in the re-import of galactose into the cell for further metabolism. In addition, many plasma membrane-localized monosaccharide transporters were expressed in the sink tissues, suggesting that these transporters might participate in phloem unloading and supply energy and monosaccharide to sink tissues [58]. *OsSWEET5* was also expressed in the stem, root and floral organs, which indicates that OsSWEET5 may function in the mobilization of galactose reserves into these tissues.

### Overexpression of OsSWEET5 causes galactose accumulation and disordered sugar distribution

The cell wall is a storage reserve of carbon for the plant body and responds to abnormal circumstances, which leads to the modification of cell wall polysaccharides, and the resulted sugars are imported into the cell for further metabolism [28], [59]. The expression of *β-lactase2* was up-regulated in the leaves of OX2 (Figure 5B). Since β-lactase is involved in the breakdown of polysaccharide to generate free β-D-Gal, the up-regulated expression of *β-lactase2* indicated that cell wall reconstruction was accelerated in transgenic plants to release galactose. In addition, the expression levels of three key genes of Leloir salvage pathway (*GalM*, *GalK* and *GalT*) were significantly altered in OX2, and *β-lactase2* and *GalM4* showed higher degree of increase in expression level compared with *GalK1* and *GalT*, leading to the accumulation of galactose (Figure 5A, B).

Sugar distribution in the whole plant plays an important role in the carbohydrate transport for sink tissues, which is important for the plant growth, and disordered sugar distribution will lead to abnormal growth in plants [8], [60]. Our study showed that compared with the leaves of the WT plants, OX2 leaves displayed sharply increased level of monosaccharides (Figure 5A) and significantly lower expression levels of *OsTMT1* and *OsTMT2* (Figure 5C). This result suggested that the transport of monosaccharides from the cytosol to the vacuole lumen was not accelerated. Moreover, the level of sucrose was significantly decreased (Figure 5A). The decreased level of sucrose may cause a lower level of sucrose transport from the source to the sink in OX2 plants. The most obvious effect was the reduced expression of *OsSUT1* and *OsSUT2* (Figure 5C), suggesting that the lower level of sucrose transport from source to sink in OX2 plants reduced the metabolic flux and thereby led to the phenotype of growth retardation (Figure 4A). The growth retardation phenotype of the transgenic plants was similar to the phenotypes of *atsweet11atsweet12* and *ossut2* [8], [33]. These results indicated that the carbon partitioning at the whole plant level was disordered, which led to the abnormal growth of the transgenic plants. In addition, sugar has hormone-like functions in regulating many genes and modulates plant growth and development similarly to phytohormones [10], and different sugar signals are generated by photosynthesis and carbon metabolism in source and sink tissues in plants [61]. Hence, all or part of phenotypes of the *OsSWEET5*-overexpressing transgenic plants might be ascribed to the disordered sugar distribution.

### Galactose accumulation and sugar distribution result in the inhibition of auxin signaling and translocation

It has been reported that galactose is an important component of the xyloglucans in the primary cell wall [62]. Nevertheless, free galactose has severe inhibitory effects on certain aspects of plant growth and development even at very low concentrations [21], [57], [63], and its inhibitory effect on auxin-induced growth could be explained by the inhibition of IAA transport [57]. In addition, excessive galactose in plants can inhibit the auxin biosynthesis and translocation directly or indirectly, and even induce leaf darkening or chlorosis and growth arrest [18]. In this study, no significant difference was observed in the expression levels of the genes involved in ethylene biosynthesis between the OX2 and WT plants (data not shown), which indicates that the phenotypes of OX2 plants were not caused by the accumulation of ethylene. Nevertheless, the free IAA level was significantly decreased in the OX2 plants with reduced transcripts of auxin signaling and translocation genes including *OsPIN1*, *IAA24* and *IAA31* (Figure 6). In addition, the expression levels of *OsGH3-2* and *SAUR39* were markedly increased in OX2 compared with in WT (Figure 6B). It has been reported that *OsGH3-2* encodes an IAA-amido synthetase and inactivates IAA by conjugating it to amino acids to suppress auxin signaling in rice, and the activation of *OsGH3-2* promoted the formation of IAA-amido, resulting in a decrease in the free IAA level in the *OsGH3-2* overexpressing lines [46], [64]. SAUR39 acts as a negative regulator of auxin synthesis and transport in rice, and *SAUR39*-overexpressing plants displayed reduced growth rate and earlier senescence progression compared with WT plants [14]. These results suggest that auxin signaling and translocation have been inhibited to retard the growth of the *OsSWEET5*-overexpressing plants.

Sugars can regulate many important processes which are also controlled by hormones including auxin during plant growth and development [65]. The crosstalk of sugar and auxin was also observed in the OX2 plants in our work. In this study, the expression levels of *Inv1* and *Sus* were increased in OX2 compared with in WT (Figure 5B), suggesting that the hydrolysis of Inv- and Sus-mediated sucrose degradation was accelerated. On one hand, the ratio of hexose to sucrose is an important factor in the regulation of IAA biosynthesis [12]. Hence, the lower IAA level in OX2 plants may be due to the higher ratio of hexose to sucrose (Figure 5A, 6A). On the other hand, hexoses released from Inv- or Sus-mediated sucrose degradation can modulate a variety of developmental processes through interacting with diverse pathways including hormonal regulation and PCD pathways [66], which correlates with the lower IAA and chlorophyll levels in OX2 plants (Figure 4B, 6A). In addition, the phenotypes in OX2 plants in our work were in conformity with the findings reported previously. As has been reported, an inverse relation between a lower auxin level and higher sugar content has been observed in Arabidopsis [10]. A lower auxin level would lead to an increased sugar level, which would repress the expression of photosynthetic genes and chlorophyll production, and eventually cause the growth retardation phenotypes including leaf senescence and smaller shoot and root [14], [15].

### Knockout of OsSWEET5 causes no obvious phenotypes

Since *OsSWEET5*-overexpressing plants displayed growth retardation phenotype, we generated knockout lines using amiRNA method (Methods S2) to further explore the function of *OsSWEET5* in rice. The expression of *OsSWEET5* was significantly suppressed in *amiRNA*-*OsSWEET5* transgenic lines (Figure S5A). However, no obvious phenotype was observed in the *amiRNA*-*OsSWEET5* transgenic lines (Figure S5B, C). The results of qRT-PCR showed that the transcript levels of genes involved in galactose metabolism, sucrose metabolism and transport were not changed in *amiRNA*-*OsSWEET5* transgenic lines with the exception of *Sus1* (Figure S5D, E). One possible explanation for this is that there might be an abundance of galactose transporters in rice. Indeed, the expression of *AtSWEET13* was significantly induced in the *atsweet11atsweet12* double mutant compared with in the WT plants [33], suggesting that SWEET genes are functionally redundant [67]. Hence, there might be other sugar transporters which can complement the galactose transport in the *amiRNA*-*OsSWEET5* plants.

## Conclusions

In summary, we identified a galactose transporter gene named *OsSWEET5* in rice. The *OsSWEET5*-overexpressing plants showed the phenotypes of growth retardation, precocious senescing leaves and changed sugar content with a reduced auxin level at seedling stage. These phenotypes might be attributed to the sugar and auxin crosstalk. The results of the present study will facilitate a better understanding on the roles of OsSWEET5 as a galactose transporter in the growth regulation of rice. Further studies on the silencing of double/multiple genes might help to elucidate the roles of *OsSWEET5* and thus to delineate how the crosstalk between sugar and auxin modulates rice growth and development. In addition, optimizing the expression of sugar transport genes is advantageous for carbohydrates transport and reserve, which may greatly facilitate the genetic improvement of yield in rice.

## Supporting Information

Figure S1 **Sequence alignment of MtN3 family proteins using the Clustal_X program.** The predicted MtN3 domains were denoted by underline. The accession numbers of these proteins are as follows: OsSWEET5 (NP_001056475), *Sorghum bicolor* (XP_002441609), *Brachypodium distachyon* (XP_003576074), OsSWEET7c/xa25 (Q2QWX8), *Zea mays* (NP_001149011), *Vitis vinifera* (XP_002283068), *Solanum lycopersicum* (CAE47557), *Arabidopsis thaliana* (XP_002877087), *Glycine max* (XP_003553885), *Ricinus communis* (XP_002518862), *Populus trichocarpa* (XP_002304566), *Medicago truncatula* (XP_003601464), Os8N3/xa13 (NP_001062354), OsSWEET14/Os11N3 (NP_001067955).
(TIF)

Figure S2 **Expression pattern of** ***OsSWEET5*** **.** qRT-PCR analysis of *OsSWEET5* transcript levels in root at seedling with 2 tillers (R1), leaf at secondary branch primordium stage (L1), 4–5 cm young panicle (P1), flag leaf at 5 days before heading (L2), stem at heading stage (S), panicles at heading stage (P2), lemma at 1 day before flowering (Le), rachis at 1 day before flowering (Ra), stamen at 1 day before flowering (St), pistil at 1 day before flowering (Pi), lodicule at 1 day before flowering (Lo), root at 1 day before flowering (R2), endosperm at 14 days after pollination (En), and flag leaf at 14 days after heading (L3), respectively. Error bars indicate standard deviation of three independent experiments. *Actin1* was used as a control for normalization.
(TIF)

Figure S3 **OsSWEET5 protein is predicted by TMHMM to contain seven transmembrane helices.**
(TIF)

Figure S4 **Southern blot analysis of the copy number of** ***OsSWEET5*** **-overexpressing plants.** M: λ-*Eco*T14 I digest DNA marker. Line 1 to 21, *OsSWEET5*-overexpressing transgenic plants. The single copy insert plants were marked with asterisk.
(TIF)

Figure S5 ***AmiRNA-OsSWEET5*** **transgenic plants had no significant differencecompared with WT plants.** (A) Expression level of *OsSWEET5* in two transgenic lines and WT examined by RT-PCR. RNA was extracted from panicles of two transgenic lines and WT at flowering stage. *Actin1* was used as an internal control. (B) Photograph of two transgenic lines and WT at tillering stage. (C) Measurement of chlorophyll content in the second leaves of transgenic plants and WT at tillering stage. Values are the means ± SD (*n* = 3). (D–E) The expression of genes involved in sugar metabolism and transport in two transgenic lines and WT plants using qRT-PCR. The first-strand cDNAs were prepared using RNAs isolated from the second leaves of two transgenic lines and WT at tillering stage. Bar represents mean (3 replicates) ± standard deviation.
(TIF)

Methods S1 **Southern blot analysis.**
(DOC)

Methods S2 **AmiRNA construction and rice transformation.**
(DOC)

Table S1 **Primers used in this study.**
(XLS)

Table S2 **Predicting structures of transmembrane helices in OsSWEET5 using SOSUI.**
(XLS)
