# Supplementary figures and images for: Overexpression of OsSWEET5 in Rice Causes Growth Retardation and Precocious Senescence
Source: PLoS One. 2014 Apr 7;9(4):e94210. doi: 10.1371/journal.pone.0094210 (PMC3978035; doi:10.1371/journal.pone.0094210)

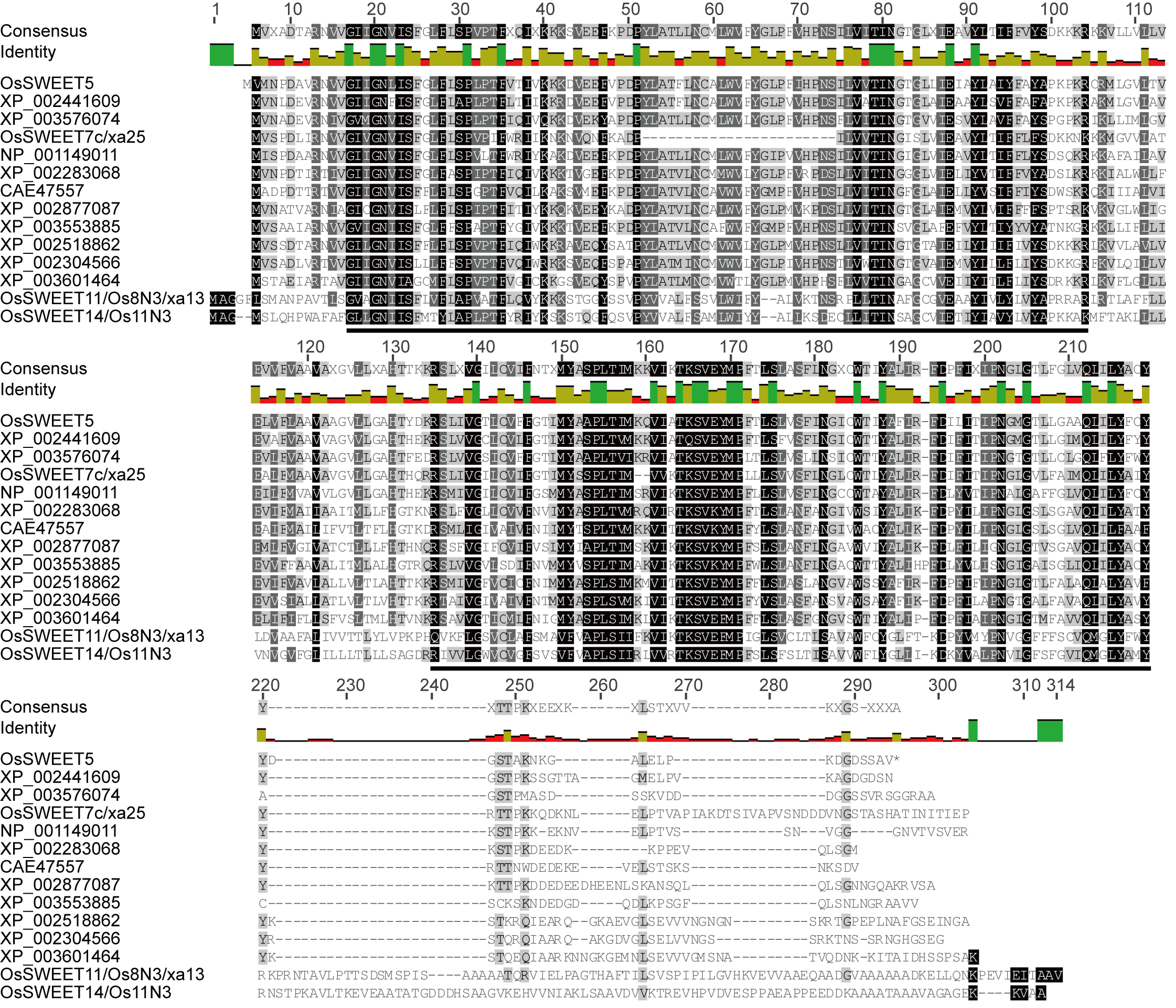

Supplement: Figure S1 — Sequence alignment of MtN3 family proteins using the Clustal_X program. The predicted MtN3 domains were denoted by underline. The accession numbers of these proteins are as follows: OsSWEET5 (NP_001056475), Sorghum bicolor (XP_002441609), Brachypodium distachyon (XP_003576074), OsSWEET7c/xa25 (Q2QWX8), Zea mays (NP_001149011), Vitis vinifera (XP_002283068), Solanum lycopersicum (CAE47557), Arabidopsis thaliana (XP_002877087), Glycine max (XP_003553885), Ricinus communis (XP_002518862), Populus trichocarpa (XP_002304566), Medicago truncatula (XP_003601464), Os8N3/xa13 (NP_001062354), OsSWEET14/Os11N3 (NP_001067955). (TIF) [file pone.0094210.s001.tif]

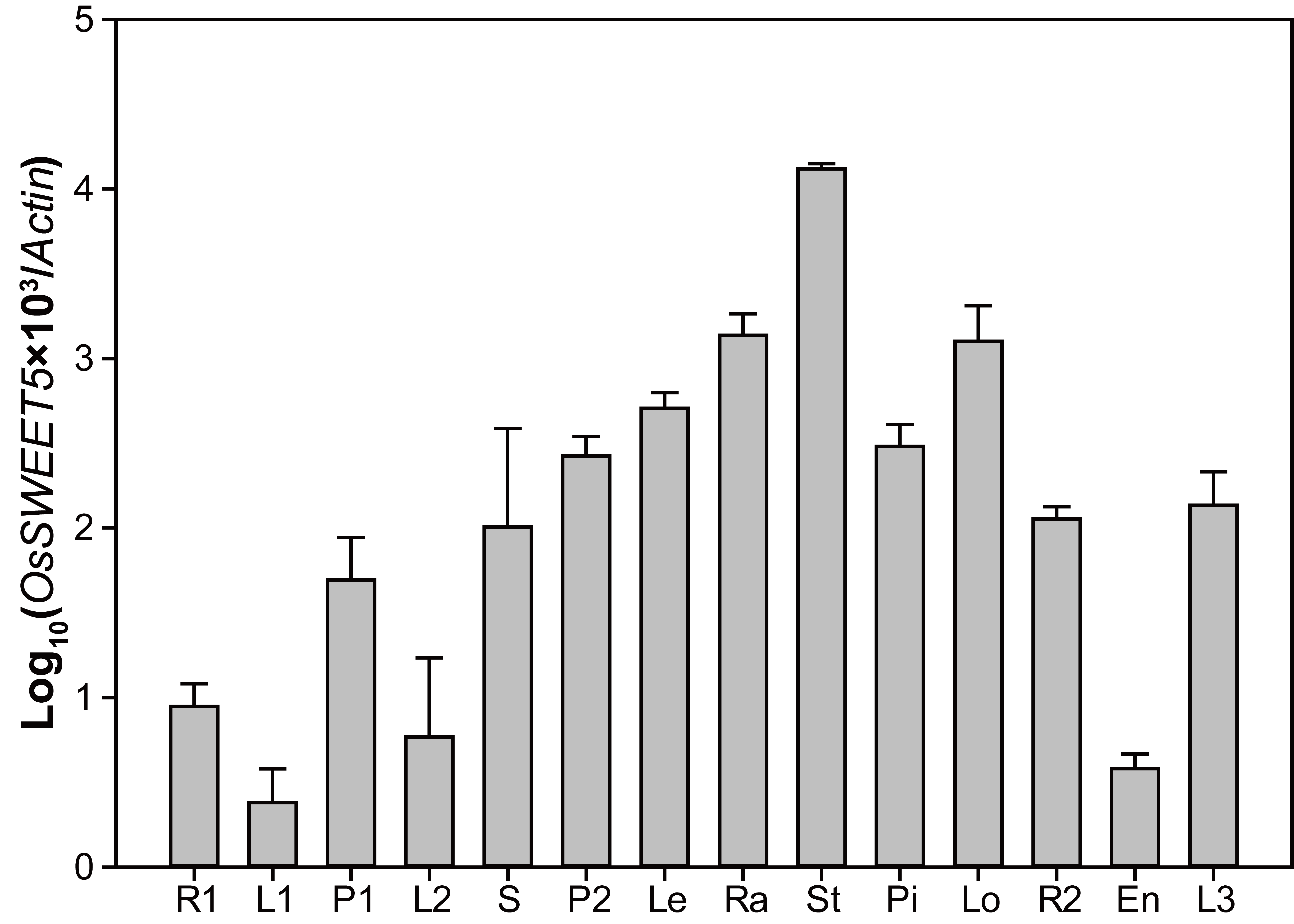

Supplement: Figure S2 — Expression pattern of OsSWEET5 . qRT-PCR analysis of OsSWEET5 transcript levels in root at seedling with 2 tillers (R1), leaf at secondary branch primordium stage (L1), 4–5 cm young panicle (P1), flag leaf at 5 days before heading (L2), stem at heading stage (S), panicles at heading stage (P2), lemma at 1 day before flowering (Le), rachis at 1 day before flowering (Ra), stamen at 1 day before flowering (St), pistil at 1 day before flowering (Pi), lodicule at 1 day before flowering (Lo), root at 1 day before flowering (R2), endosperm at 14 days after pollination (En), and flag leaf at 14 days after heading (L3), respectively. Error bars indicate standard deviation of three independent experiments. Actin1 was used as a control for normalization. (TIF) [file pone.0094210.s002.tif]

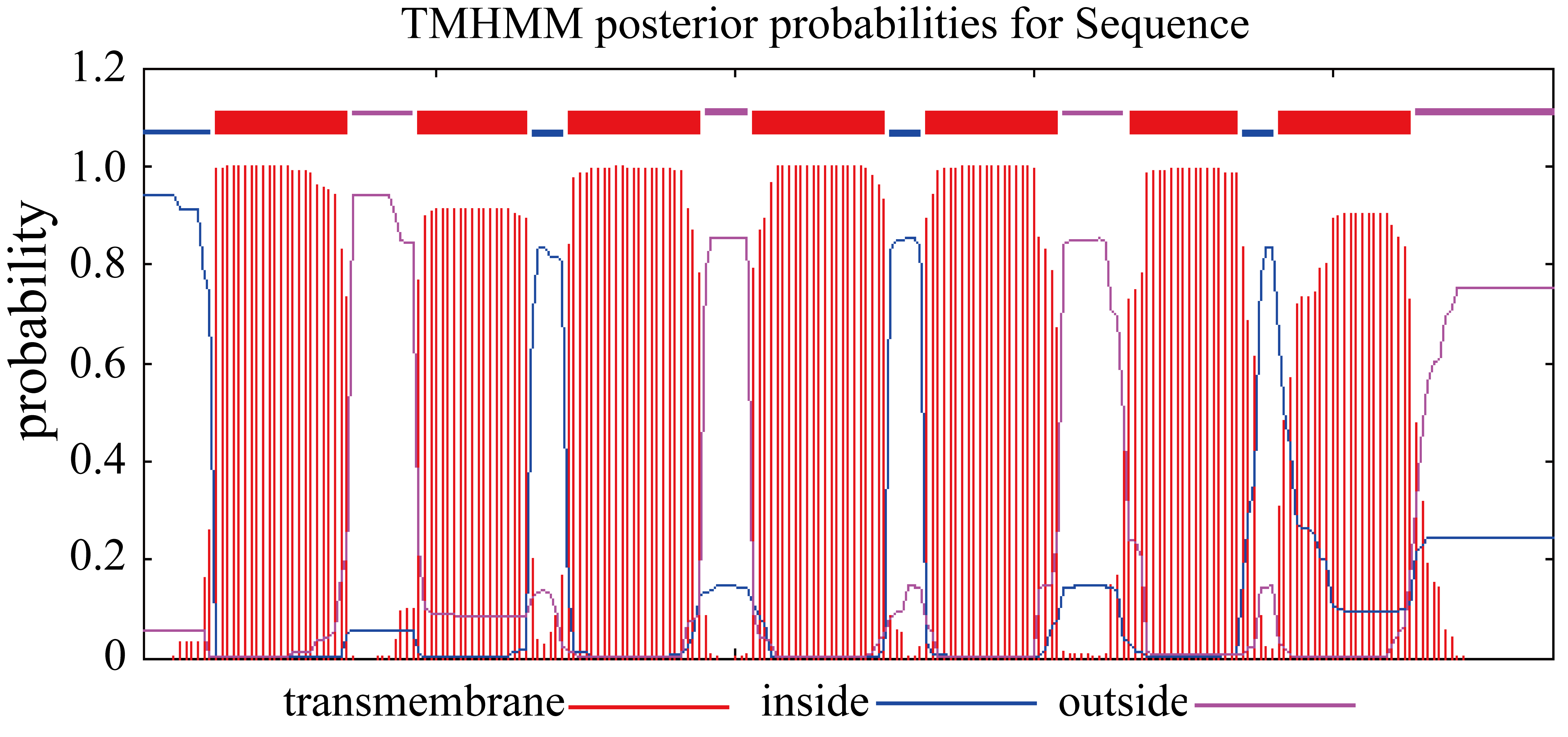

Supplement: Figure S3 — OsSWEET5 protein is predicted by TMHMM to contain seven transmembrane helices. (TIF) [file pone.0094210.s003.tif]

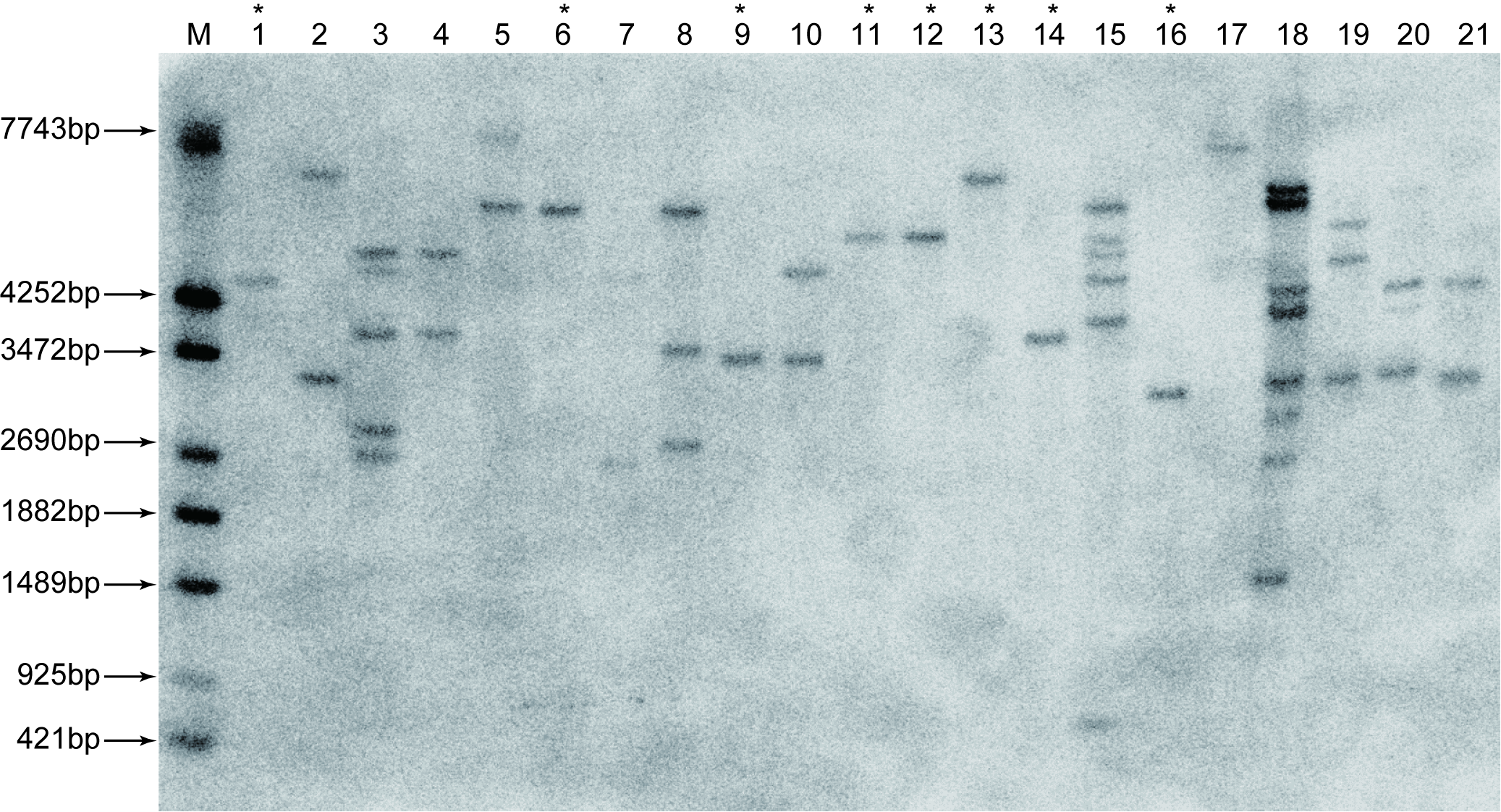

Supplement: Figure S4 — Southern blot analysis of the copy number of OsSWEET5 -overexpressing plants. M: λ-EcoT14 I digest DNA marker. Line 1 to 21, OsSWEET5-overexpressing transgenic plants. The single copy insert plants were marked with asterisk. (TIF) [file pone.0094210.s004.tif]

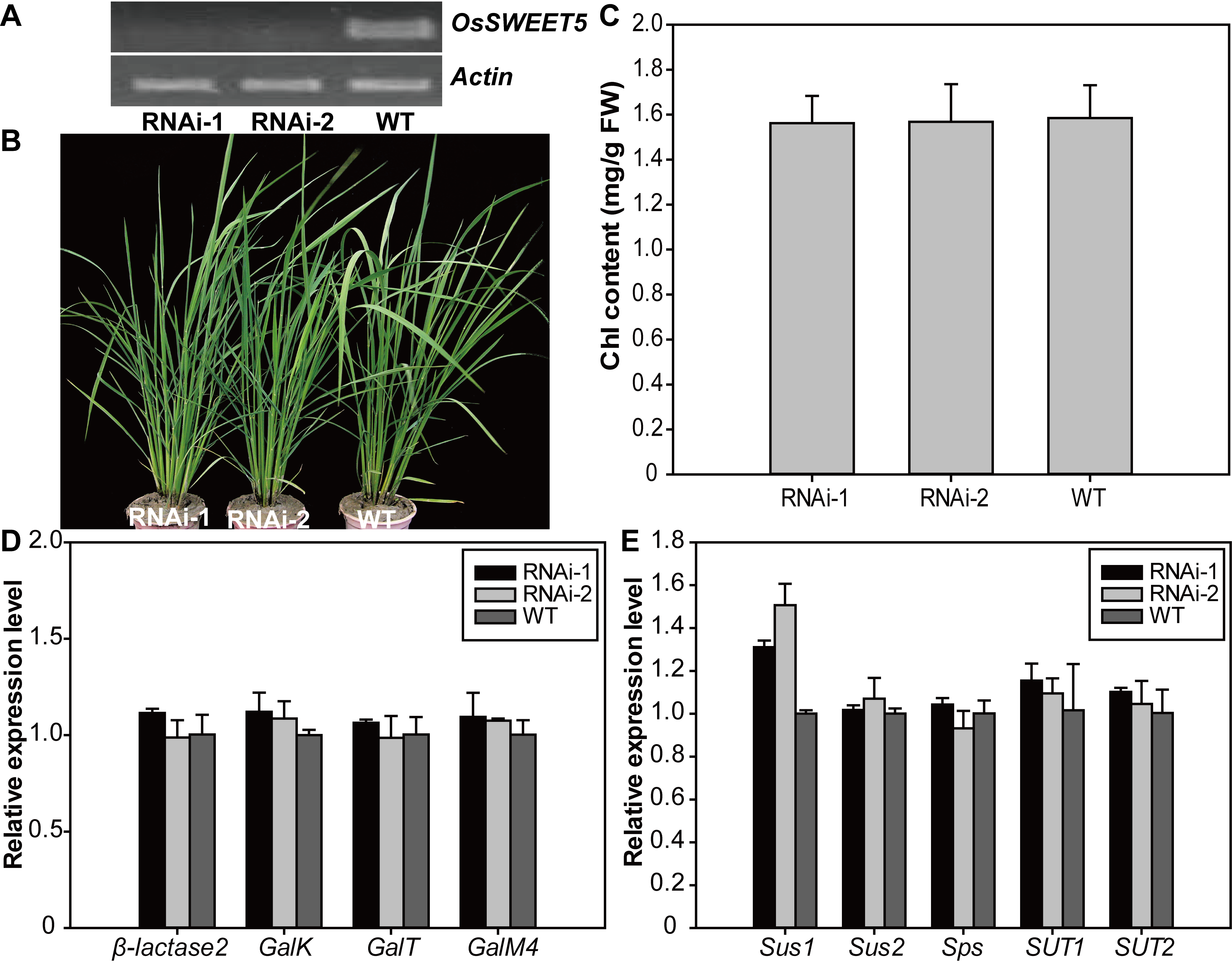

Supplement: Figure S5 — AmiRNA-OsSWEET5 transgenic plants had no significant differencecompared with WT plants. (A) Expression level of OsSWEET5 in two transgenic lines and WT examined by RT-PCR. RNA was extracted from panicles of two transgenic lines and WT at flowering stage. Actin1 was used as an internal control. (B) Photograph of two transgenic lines and WT at tillering stage. (C) Measurement of chlorophyll content in the second leaves of transgenic plants and WT at tillering stage. Values are the means ± SD (n = 3). (D–E) The expression of genes involved in sugar metabolism and transport in two transgenic lines and WT plants using qRT-PCR. The first-strand cDNAs were prepared using RNAs isolated from the second leaves of two transgenic lines and WT at tillering stage. Bar represents mean (3 replicates) ± standard deviation. (TIF) [file pone.0094210.s005.tif]
