# Supplementary material for: Overexpression of OsSWEET5 in Rice Causes Growth Retardation and Precocious Senescence
Source: PLoS One. 2014 Apr 7;9(4):e94210. doi: 10.1371/journal.pone.0094210 (PMC3978035; doi:10.1371/journal.pone.0094210)
Supplement: Methods S1 — Southern blot analysis. (DOC) [file pone.0094210.s006.doc]

**Methods S1. Southern blot analysis**

The positive independent *OsSWEET5*-overexpressing transgenic plants were further confirmed by Southern blot. Total DNA from fresh leaf tissue was isolated, digested with appropriate restriction enzyme and then transferred to Hybond nylon membrane (Amersham, USA), hybridized with ^32^P-labeled specific probe, and the hybridization signal was captured by Fujifilm FLA-5100 (Fujifilm, Japan) as previously described . The probe was amplified by PCR using a Random Primer DNA Labeling Kit (TaKaRa, Japan) with hpt-specific primers listed in Table S1. The PCR program was as follows: 94°C for 5 min, followed by 30 cycles of 94°C for 30 s, 58°C for 30 s, and 72°C for 30 s, and finally 72°C for 7 min.

# References

1. Lin Y, Zhang Q (2005) Optimising the tissue culture conditions for high efficiency transformation of indica rice. Plant Cell Rep 23: 540-547.
2. Du H, Liu L, You L, Yang M, He Y, et al. (2011) Characterization of an inositol 1, 3, 4-trisphosphate 5/6-kinase gene that is essential for drought and salt stress responses in rice. Plant Mol Biol 77: 547-563.
