# Supplementary material for: Overexpression of OsSWEET5 in Rice Causes Growth Retardation and Precocious Senescence
Source: PLoS One. 2014 Apr 7;9(4):e94210. doi: 10.1371/journal.pone.0094210 (PMC3978035; doi:10.1371/journal.pone.0094210)
Supplement: Methods S2 — AmiRNA construction and rice transformation. (DOC) [file pone.0094210.s007.doc]

**Methods S2. A****miRNA construction and rice transformation**

For the amiRNA construction, the amiRNA sequence was designed by Web MicroRNA Designer (<http://wmd3.weigelworld.org/cgi-bin/webapp.cgi>) and four primers were obtained (OsSWEET5a, OsSWEET5b, OsSWEET5c and OsSWEET5d). Three modification PCRs were performed with primers G-4368+OsSWEET5b, OsSWEET5a+OsSWEET5d and OsSWEET5c+G-4369 using pNW55 as a template, and the three PCR fragments were subsequently mixed as a template for PCR with G-4368 and G-4369. The PCR product was digested with K*pn* I and B*am*H I and cloned into IRS154 as described previously . The PCR program was as follows: 95°C for 2 min, followed by 30 cycles of 95°C for 30 s, 55°C for 30 s, and 72°C for 1 min, 72°C for 7 min. The sequences of primers are listed in Table S1. AmiRNA construct was transformed into Zhonghua 11 as previously described .

# References

1. Warthmann N, Chen H, Ossowski S, Weigel D, Hervé P (2008) Highly specific gene silencing by artificial miRNAs in rice. PLoS One 3: e1829.
2. Liu L, Zhou Y, Szczerba MW, Li X, Lin Y (2010) Identification and application of a rice senescence-associated promoter. Plant Physiol 153: 1239-1249.
